# Computable phenotypes to identify respiratory viral infections in the *All of Us* research program

**DOI:** 10.1038/s41598-025-02183-9

**Published:** 2025-05-28

**Authors:** Bennett J. Waxse, Fausto Andres Bustos Carrillo, Tam C. Tran, Huan Mo, Emily E. Ricotta, Joshua C. Denny

**Affiliations:** 1 National Human Genome Research Institute, National Institutes of Health, Bethesda, MD USA; 2 National Institute of Allergy and Infectious Diseases, National Institutes of Health, Bethesda, MD USA; 3 Department of Preventive Medicine and Biostatistics, Uniformed Services University of the Health Sciences, Bethesda, MD USA; 4 All of Us Research Program, National Institutes of Health, Bethesda, MD USA

**Keywords:** Electronic health records, Computable phenotype, Respiratory tract infections, Influenza virus, Reproducibility of results, Precision medicine, Viral infection, Bioinformatics, Data integration

## Abstract

Electronic health records (EHRs) contain rich temporal data about respiratory viral infections, but methods to identify these infections from EHR data vary widely and lack robust validation. We developed computable phenotypes by integrating virus-specific International Classification of Diseases (ICD) billing codes, prescriptions, and laboratory results within 90-day episodes. Analysis of 265,222 participants with EHR data from the *All of Us* Research Program yielded national cohorts of varied size: large cohorts for SARS-CoV-2 (*n* = 28,729) and influenza (*n* = 19,784); medium cohorts for rhinovirus, human coronavirus, and respiratory syncytial virus (*n* = 1,161-1,620); and smaller cohorts for the other viruses (*n* = 238–486). Using laboratory results as a reference standard, phenotypes using virus-specific ICD codes and medications had variable sensitivity (8–67%) but high positive predictive value (PPV, 90–97%) for most viruses, while influenza virus and SARS-CoV-2 phenotypes had lower PPV (69–70%) that improved with the inclusion of additional ICD codes. Identified infections exhibited expected seasonal patterns matching CDC data. This integrated approach identified infections more effectively than individual components alone and demonstrated utility for severe infections in hospital settings. This method enables large-scale studies of host genetics, health disparities, and clinical outcomes across episodic diseases, with flexibility to optimize sensitivity or PPV depending on the specific research question.

**Supplementary Information:**

The online version contains supplementary material available at 10.1038/s41598-025-02183-9.

## Introduction

Respiratory infections are among the most common human diseases, with over 25 known viruses capable of causing respiratory tract disease^1^. While research has traditionally focused on influenza virus, respiratory syncytial virus (RSV), and recently SARS-CoV-2, multiplex diagnostic testing has revealed that rhinovirus (RV), human metapneumovirus (hMPV), parainfluenza viruses (PIV), and common human coronaviruses (hCoVs) can lead to comparable clinical presentations, detection frequencies, and healthcare utilization in hospitalized patients^2–6^. Despite their clinical impact and burden, these less frequently identified respiratory pathogens remain understudied.

Population-level studies of respiratory infections face significant methodological challenges. Research typically relies on administrative claims data, laboratory surveillance, or curated clinical cohorts^5,7^. While laboratory results and some pathogen-specific International Classification of Diseases (ICD) codes are highly specific, they have poor sensitivity due to infrequent testing and identification. In pediatric patients, for instance, fewer than 10% who present with influenza-like illness or upper respiratory infection receive laboratory testing during standard care, and more than half receive only nonspecific syndromic ICD diagnoses^8,9^. Testing rates also vary by age, season, illness severity, length of stay, and rurality, creating biases in who receives diagnostic confirmation^9^. Even for common viruses like influenza virus and RSV, adding non-specific ICD codes only modestly improves sensitivity and dramatically reduces positive predictive value^10,11^. Further complicating these efforts, ICD code sets used in respiratory infection research are rarely shared and even less frequently validated using laboratory results or other samples^12,13^.

Electronic health record (EHR)-based phenotyping algorithms that can integrate multiple data types offer a promising approach to reliably construct disease cohorts for observational research. While combining billing codes, clinical notes, and medications using EHRs improves performance for many diseases, this approach has rarely been applied for respiratory viruses^5,14–17^. Developing and validating such algorithms for respiratory viruses would enable studies of clinical, environmental, and genetic risk factors across many respiratory viruses within existing biobanks.

This study aimed to create and assess computable phenotypes for identifying viral respiratory infections in EHR data using the National Institutes of Health’s *All of Us* Research Program (*All of Us*). *All of Us* collects a variety of participant data including surveys, physical measurements, biospecimens, and EHRs. We constructed computable phenotypes by integrating virus-specific ICD codes, antiviral medications, and laboratory results. To assess phenotype performance, we calculated specificity, positive predictive value (PPV) and sensitivity for combinations of phenotype components (ICD codes with different count thresholds and medications) using laboratory testing as a reference standard. We evaluated the reliability of our approach by comparing the frequency and distribution of laboratory results against CDC surveillance data.

## Results

**Fig. 1:**
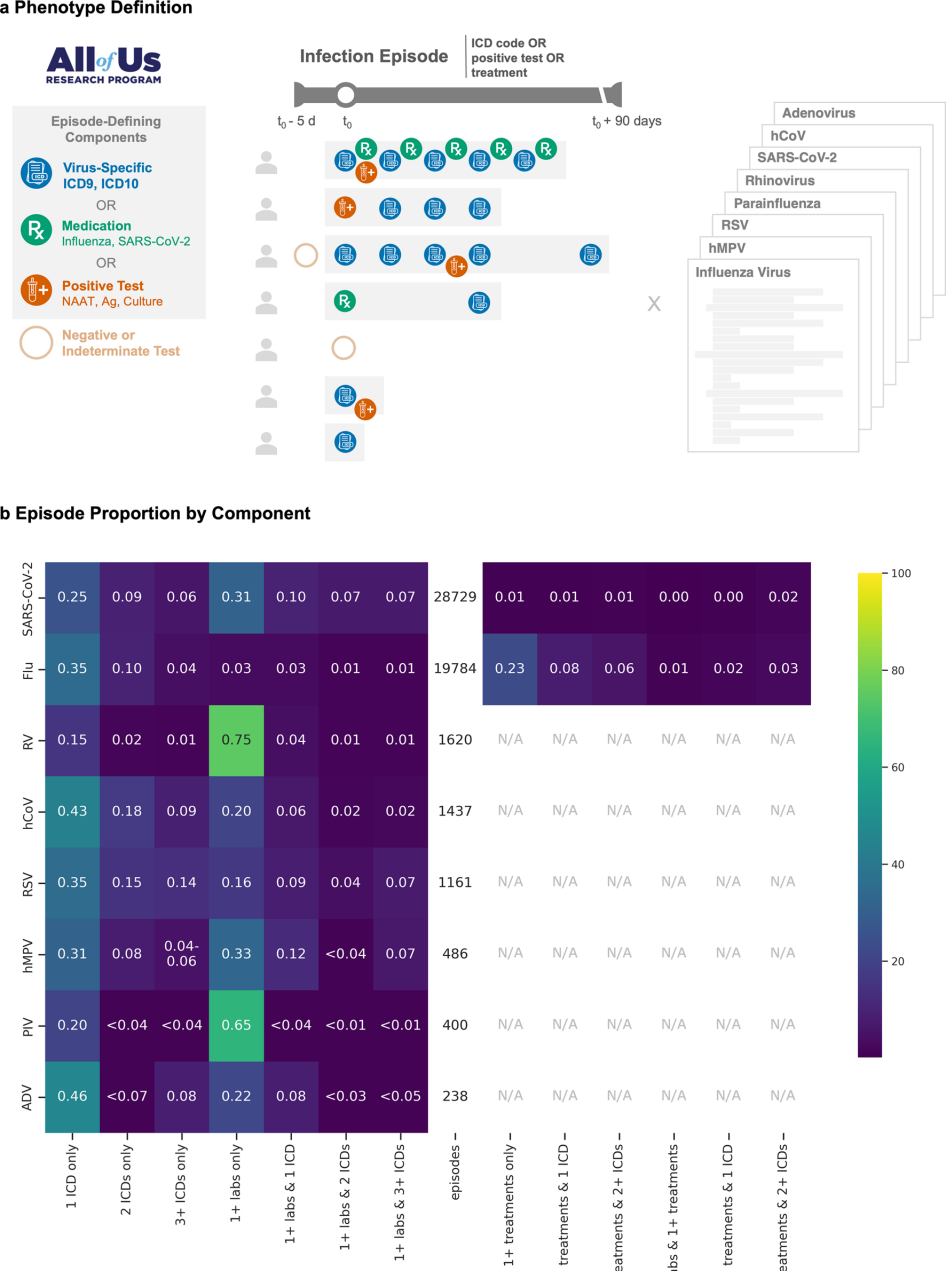
Computational phenotype for respiratory virus episodes using electronic health records (EHRs) with episode composition. (**a**) Episodes were defined by (1) virus-specific ICD-9-CM or ICD-10-CM codes, (2) antiviral medications (for influenza and SARS-CoV-2), and/or (3) positive laboratory results including nucleic acid amplification tests (NAAT), antigen tests, or cultures. The first qualifying event is designated as time zero (t_0_), and all related, subsequent events within 90 days were grouped into the same episode. Negative or indeterminate tests were also included, with a five-day lookback window to incorporate false negative results. Phenotypes were computed for influenza virus, human metapneumovirus (hMPV), respiratory syncytial virus (RSV), parainfluenza, rhinovirus (RV), SARS-CoV-2, common human coronavirus (hCoV), and adenovirus (ADV). Heatmap (**b**) showing the breakdown of episode types (columns) for each virus (row). Colors indicate the percentage of total episodes for each virus (e.g., 74.9% of RV episodes (1,210/1,620) contained only positive laboratory results. Percentages and corresponding colors for counts below 20, were censored per the *All of Us* participant privacy policy. N/A: not applicable, as these viruses do not have regularly used treatments.

We analyzed EHR data from 265,222 *All of Us* participants between 1981 and 2022, developing computable phenotypes for eight respiratory viruses: rhinovirus (RV), human metapneumovirus (hMPV), respiratory syncytial virus (RSV), adenovirus (ADV), SARS-CoV-2, parainfluenza (PIV), common human coronavirus (hCoV), and influenza virus. Patient encounters were identified in the EHR if they had a virus-specific ICD code (e.g., ICD-9-CM 487 “influenza” or ICD-9-CM 487.0 “Influenza with pneumonia”), a positive laboratory test, or an antiviral prescription (for influenza and SARS-CoV-2 only). All virus-specific ICD codes, laboratory results, and medications used for phenotyping are provided in Tables S1-3. All subsequent related events within 90 days were grouped into the same illness episode (Fig. 1a)^5^.

### Cohort characteristics

We identified respiratory virus episodes that varied substantially in size and composition (Fig. 1b). The largest cohorts were SARS-CoV-2 (*n* = 28,729 distinct episodes) and influenza virus (*n* = 19,784); followed by RV (*n* = 1,620), hCoV (*n* = 1,437), RSV (*n* = 1,161); and the smaller cohorts, hMPV (*n* = 486), PIV (*n* = 400), and ADV (*n* = 238). EHR data availability varied by virus, with the earliest records dating back to 1981 (influenza virus), followed by 1987 (ADV), 1997 (RSV), 2002 (PIV, hMPV), 2003 (RV), 2012 (hCoV), and 2020 (SARS-CoV-2).

Across all cohorts, participants were predominantly female (61–68%) with median ages mostly between 50 and 58 (Table S4). Participants who self-reported as White were the plurality for every virus (32.9–60.1%), compared to participants self-reporting as Black (16.5–28.5%) or Hispanic/Latino (17-32.1%). All other options (Asian, multiple selected, Middle Eastern or North African, and Native Hawaiian or Other Pacific Islander) were rare (0-2.2%). SARS-CoV-2 and influenza virus participant demographics most closely mirrored the overall *All of Us* cohort with ICD, laboratory, or medication data (Table S4). Compared to all other groups, SARS-CoV-2 and influenza virus cohorts had a higher proportion of participants who self-reported as White (50.4–60.1%), and they more frequently reported a higher income, education, and employer-provided insurance. Demographic data were only notably missing for insurance type (46,487/265,222 = 17.5% for all participants with EHR data). For each virus, participants identified as a viral case by the phenotype algorithm (‘infected’ in Table S4) received more tests per person compared to participants ever tested for that virus (‘tested’ in Table S4).

Analysis of episode composition revealed differences between viruses. Some viruses primarily consisted of laboratory results alone (predominantly for RV [74.7%], PIV [65.0%], SARS-CoV-2 [31.3%], hMPV [32.9%]) or single ICD codes (predominantly for ADV [45.8%], hCoV [43.1%], RSV [35.4%], influenza [34.8%]; Fig. 1b). Antiviral use varied: SARS-CoV-2 episodes rarely included antiviral prescriptions (4.57%), while medication-only episodes were frequently observed for influenza virus (22.9%), even after excluding prophylactic prescriptions.

### Phenotype performance for detecting true positives

**Fig. 2:**
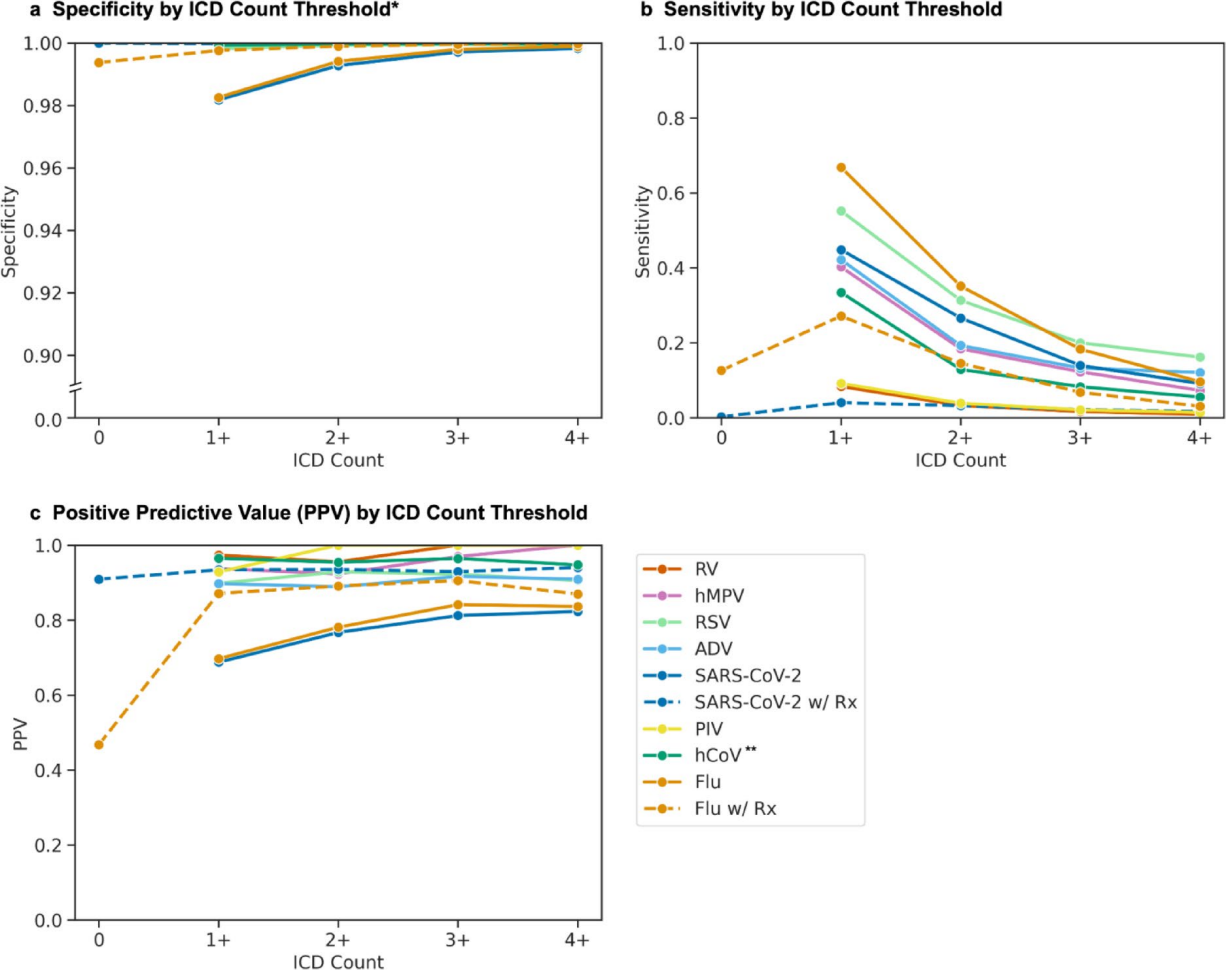
Phenotype performance across different episode definitions. Specificity (**a**), sensitivity (**b**), and positive predictive value (PPV; **c**) were calculated using nucleic acid amplification and virus culture laboratory results as the reference standard. For each virus, colored lines show performance across episodes containing increasing numbers of ICD codes. For influenza virus (orange line) and SARS-CoV-2 (dark blue line), additional dashed lines show performance when episodes were restricted to episodes containing both ICD codes and antiviral prescriptions (Rx). *Y-axis for specificity (**a**) is broken to depict differences near 1.0. **Human coronavirus (hCoV) episodes excluded ICD codes after February 1, 2020, due to loss of specificity during the COVID-19 pandemic (Figure S1a). PIV: parainfluenza; hMPV: human metapneumovirus; RV: rhinovirus; ADV: adenovirus; RSV: respiratory syncytial virus; hCoV: human coronavirus; Flu: influenza virus.

To understand how ICD code counts affected phenotype performance, we calculated sensitivity, specificity, and positive predictive value (PPV) for each virus using nucleic acid amplification and virus culture test results as a reference standard (Fig. 2). We compared phenotypes requiring at least 1, 2, 3, or 4 instances of relevant ICD code per episode. The sensitivity of using at least one virus-specific ICD code varied between viruses and decreased with the inclusion of additional codes. The sensitivity of just one ICD code was highest for influenza virus (66.8%), compared to moderate sensitivity for RSV (55.2%), SARS-CoV-2 (44.8%), ADV (42.4%), hMPV (40.2%), and hCoV (33.4%). RV (9.2%) and PIV (8.3%) were rarely identified by ICD codes alone regardless of how many appeared in an encounter.

Specificity and PPV demonstrated similar patterns, with exaggerated variation in PPV initially demonstrating three groupings. First, for influenza virus and SARS-CoV-2, the PPV for one or more ICD codes was lower (69.7% and 68.8%, respectively), but it increased as the minimum *N* ICD count increased (78.1% and 76.7% for at least 2 ICD codes, respectively; Fig. 2). Second, for other respiratory viruses except hCoV, the PPV was high (89.7–97.3%) regardless of ICD code count. Third, hCoV initially demonstrated a high PPV (79.5%) that decreased as ICD count increased (71.8% for at least 2 ICD codes; Figure S1a).

The unusual pattern for hCoV occurred during the COVID-19 pandemic, when the nonspecific hCoV ICD code counts spiked above historical maxima despite an absence of positive tests (Figure S1b). After excluding hCoV ICD codes recorded after February 1, 2020, the PPV pattern for hCoV aligned with non-influenza, non-SARS-CoV-2 viruses (Fig. 2, Figure S1a).

Adding medication use to the phenotype had varying effects on performance. As with the medication-exclusive phenotypes, specificity and PPV increased with each additional ICD code for the medication-inclusive influenza and SARS-CoV-2 cohorts. While only a small proportion (1,345/28,741 = 4.67%) of SARS-CoV-2 episodes included a prescription for remdesivir, molnupiravir, or nirmatrelvir, the addition of medication to the phenotype did increase PPV for this subset of 1,345 participants (Fig. 2). For influenza virus, medication use alone was poorly predictive (PPV = 46.8%), but combining medications with 1 ICD code improved PPV compared to 1 or more codes alone (87.1% vs. 69.7%, respectively; Fig. 2).

We further evaluated combinations of ICD codes and antiviral requirements for both influenza virus and SARS-CoV-2 and demonstrated an expected trade-off in performance (Table 1). The broadest criteria - requiring only one ICD code or a medication - maximized sensitivity (76.0% influenza virus, 45.1% for SARS-CoV-2), but this caused the highest number of false positives and the lowest PPVs (65.8% and 68.8%, respectively). For influenza virus, by requiring at least two ICD codes or a medication accompanied by an ICD code, the lower sensitivity (47.7%) was accompanied by a marked reduction in false positives (778 to 238) and increase in PPV (65.8–79.8%). Similar trends were observed for SARS-CoV-2. Despite trade-offs, the φ coefficient, which quantifies the correlation between lab results and phenotypes, was highest for the most inclusive phenotypes for both viruses.

**Table 1:** Phenotype performance

		Counts (*N*)				Phenotype Performance				
	Phenotype	TP	FP	FN	TN	Sen.	Spec.	PPV	NPV	φ
**Influenza virus**	1 + ICDs or medication	1496	778	472	32,018	0.760	0.976	0.658	0.985	0.688
	1 + ICDs	1315	572	653	32,224	0.668	0.983	0.697	0.980	0.664
	2 + ICDs or medication	1120	444	848	32,352	0.569	0.986	0.716	0.974	0.619
	2 + ICDs or (1 ICD + medication)	939	238	1029	32,558	0.477	0.993	0.798	0.969	0.600
	3 + ICDs or medication	941	339	1027	32,457	0.478	0.990	0.735	0.969	0.574
	3 + ICDs or (1–2 ICDs + medication)	760	133	1208	32,663	0.386	0.996	0.851	0.964	0.558
	2 + ICDs	691	194	1277	32,602	0.351	0.994	0.781	0.962	0.506
	3 + ICDs	360	68	1608	32,728	0.183	0.998	0.841	0.953	0.379
**SARS-CoV-2**	1 + ICDs or medication	7298	3302	8894	177,219	0.451	0.982	0.688	0.952	0.526
	1 + ICDs	7258	3298	8934	177,223	0.448	0.982	0.688	0.952	0.524
	2 + ICDs or medication	4483	1324	11,709	179,197	0.277	0.993	0.772	0.939	0.438
	2 + ICDs or (1 ICD + medication)	4443	1320	11,749	179,201	0.274	0.993	0.771	0.938	0.435
	3 + ICDs or medication	2598	546	13,594	179,975	0.160	0.997	0.826	0.930	0.345
	3 + ICDs or (1–2 ICDs + medication)	2558	542	13,634	179,979	0.158	0.997	0.825	0.930	0.342
	2 + ICDs	4309	1310	11,883	179,211	0.266	0.993	0.767	0.938	0.427
	3 + ICDs	2260	523	13,932	179,998	0.140	0.997	0.812	0.928	0.318

TP: true positive; FP: false positive; FN: false negative; TN: true negative; Sen: sensitivity; Spec: specificity; PPV: positive predictive value; NPV: negative predictive value; φ: phi coefficient (mean square contingency coefficient).

**Fig. 3:**
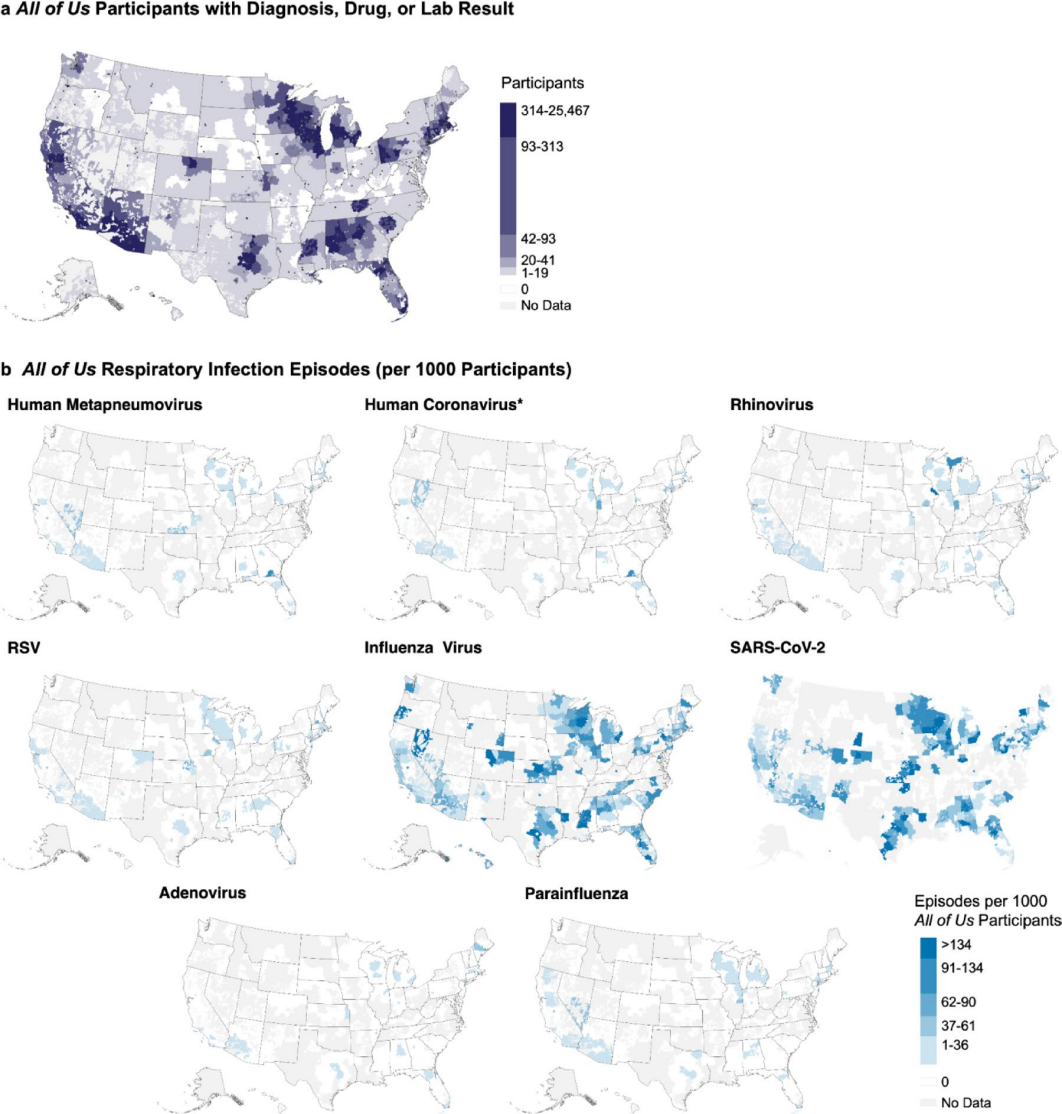
Geographic distribution of respiratory virus episodes. Heat maps show *All of Us* participants with relevant EHR data (**a**) and episode rates per 1,000 *All of Us* participants with EHR data by three-digit zip code prefix (**b**). Colors represent quintiles defined by SARS-CoV-2 rates, the largest cohort. Regions with five or fewer *All of Us* participants are marked as “No Data” in (**b**). *Human coronavirus episodes were filtered as described in methods. RSV: respiratory syncytial virus.

Geographic analysis identified broad nationwide coverage of infections, particularly for SARS-CoV-2 and influenza virus (Fig. 3). While episodes generally matched the distribution of *All of Us* participants with EHR data (Fig. 3b) and participants tested for each virus (Figure S2c), incorporating ICD codes and medications resulted in higher infection rates in the Southeast and Texas despite lower testing coverage in these regions.

**Fig. 4:**
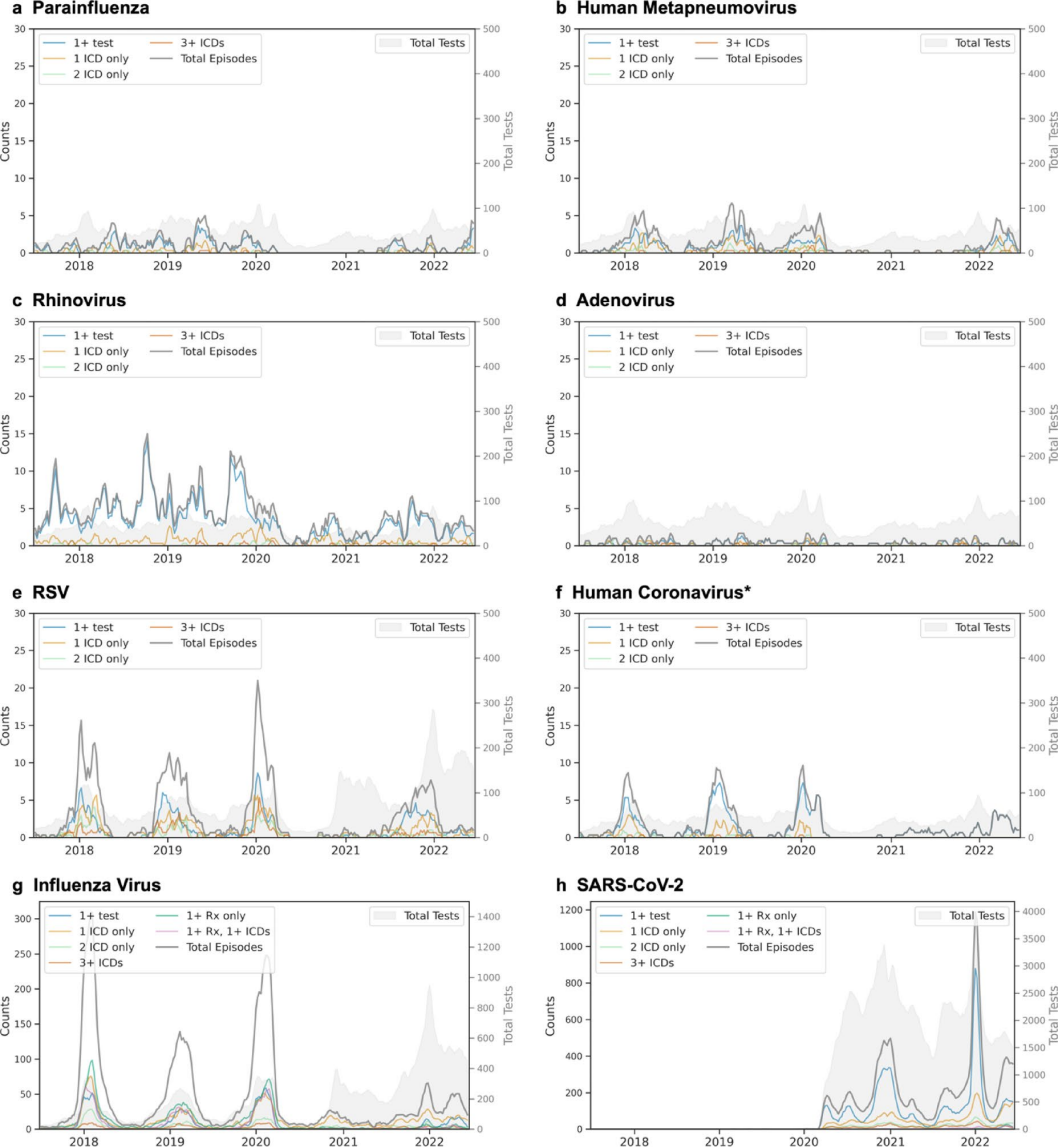
Temporal patterns of respiratory virus episodes by composition. Three-week moving averages shown for (**a**) parainfluenza, (**b**) human metapneumovirus, (**c**) rhinovirus, (**d**) adenovirus, (**e**) respiratory syncytial virus (RSV), (**f**) human coronavirus, (**g**) influenza virus, and (**h**) SARS-CoV-2. For each virus, lines show total episodes (gray) and episodes by composition: one or more positive tests (blue), single ICD code (light orange), two ICD codes (light green), and three or more ICD codes (red-orange). For influenza and SARS-CoV-2, additional lines show episodes with antiviral prescriptions alone (dark green) or with concurrent ICD codes (pink). Gray shading indicates testing volume. The left y-axis corresponds to episode counts and the right y-axis shows total tests performed. *Episodes for hCoV are shown after applying temporal filtering (unfiltered plot compared in Figure S1).

Temporal analysis showed that infection episodes composed of 1–3 ICD codes exhibited seasonality patterns consistent with laboratory test-positive episodes for all commonly detected viruses (Fig. 4). During the early COVID-19 pandemic (winter 2020 to spring 2021), only SARS-CoV-2 and RV were consistently identified.

### Patterns in phenotype composition by level of care

**Fig. 5:**
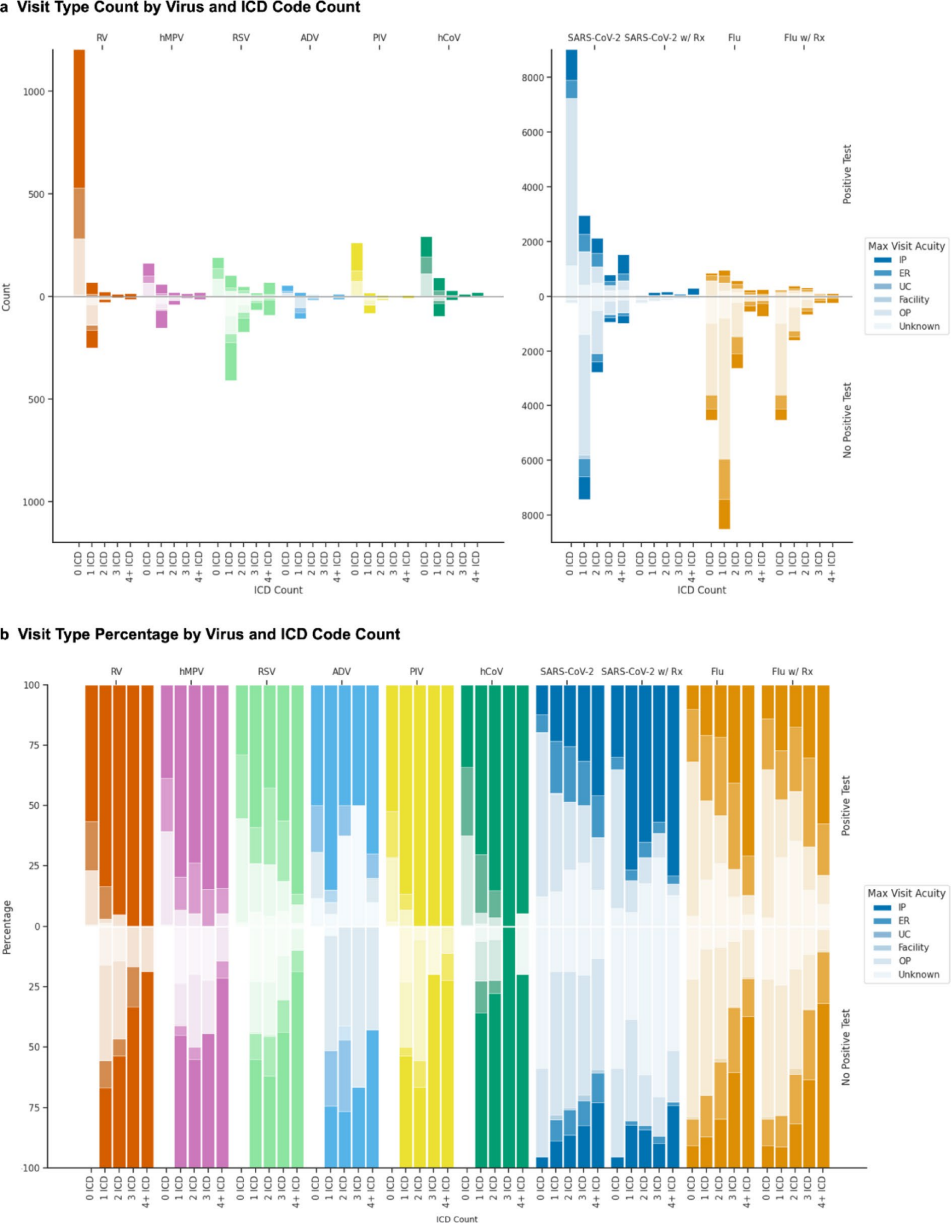
Level of care patterns by virus and episode composition. For each virus, (**a**) counts and (**b**) percentages of maximum level of care recorded between seven days before and 14 days after episode start. Results are stratified by episode characteristics: episodes with positive tests (upper panels) versus those without (lower panels), and by number of ICD codes (columns). Color intensity corresponds to level of care: inpatient (IP, darkest), emergency room (ER), urgent care (UC), outpatient (OP), and unknown (lightest). For influenza virus (Flu) and SARS-CoV-2, additional columns show episodes containing antiviral prescriptions. PIV: parainfluenza; hMPV: human metapneumovirus; RV: rhinovirus; ADV: adenovirus; RSV: respiratory syncytial virus; hCoV: human coronavirus; Rx: antiviral prescription.

Encounter level of care varied by virus and episode composition. For RV, hMPV, PIV, hCoV, and SARS-CoV-2, episodes defined by at least one test without ICD codes were the most frequent. For RSV, ADV, and influenza virus, ICD-only episodes predominated (Fig. 5). Influenza virus episodes with antiviral prescriptions were similar in the distribution of visit types compared to those without antiviral prescriptions, while SARS-CoV-2 episodes rarely included prescriptions during our study period.

By percentage, influenza virus and SARS-CoV-2 episodes included a mix of outpatient, ER, and inpatient encounters, while rates of ER visits and hospitalizations were higher for the other viruses. Episodes with positive tests had higher hospitalization rates compared to test-negative episodes, and hospitalization rates increased with the number of ICD codes per episode. The cohorts included very few post-acute care encounters and almost no urgent care encounters.

### Laboratory result comparison

Using national epidemiological data from NREVSS, COVID Data Tracker, and GISRS, we compared *All of Us* laboratory results by geographic coverage, virus type proportion, and temporal trends.

We found broad national coverage of *All of Us* participants with relevant EHR data (265,222 participants), with enriched sampling near population centers in the Northeast corridor; Western Pennsylvania; Great Lakes Region; Southeast; Arizona; California; and the metropolitan areas of Austin/Dallas, Kansas City, Denver, and Seattle (Figure S2a, Table S7). Only 3.7% of zip3 codes had no *All of Us* participants with ICD, laboratory, or medication data.

Testing patterns in the *All of Us* data overlapped with CDC clinical laboratories reporting to NREVSS (Figure S2b) and mirrored participant distribution (Figure S2a) with a notable decrease in testing for all respiratory viruses in the Southeast relative to participant density (Figure S2c). Testing frequency varied substantially by virus; participants were more frequently tested for influenza virus and SARS-CoV-2 compared to all other viruses.

Virus type distributions in *All of Us* were similar to national surveillance data from NREVSS and GISRS^18–20^. For PIV (2011–2019), HPIV-3 was most commonly detected and all other types were less frequent (Figure S3a). For hCoV (2014–2021), OC43 was most common and 229E was least common, while the order of NL63 and HKU1 differed (Figure S3b). Influenza virus type proportions (2010–2020) were nearly identical, with influenza virus A more common than influenza virus B (Figure S3c). Cross-dataset influenza subtype comparisons were not available, but in *All of Us*, H3N2 and H1N1 pdm09 were markedly more common than H1N1 and H5N1, as expected.

**Fig. 6:**
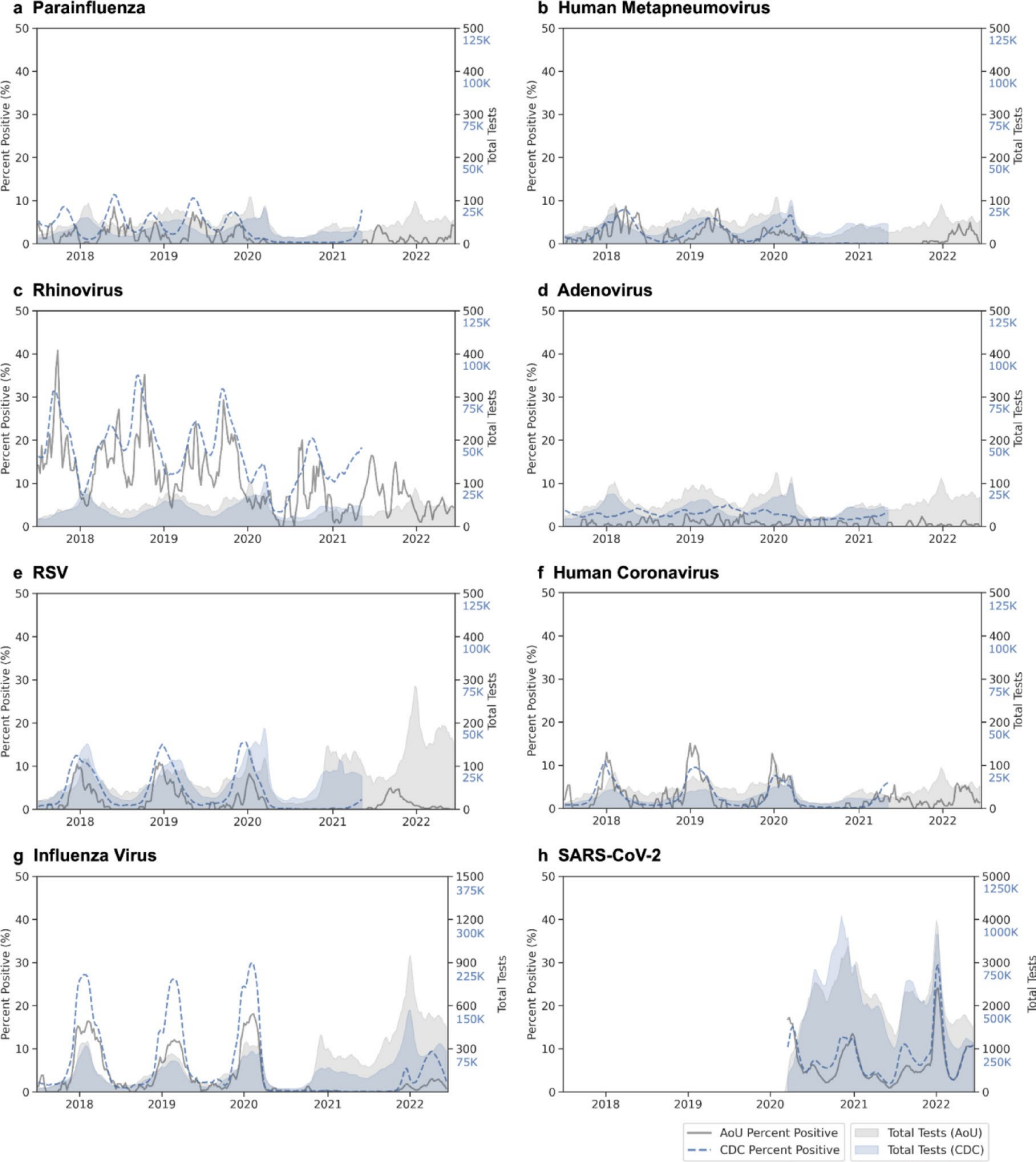
Temporal validation of test positivity using CDC surveillance data. Three-week moving average of test positivity and test volume comparing *All of Us* (gray) to CDC surveillance data (blue) for eight respiratory viruses: (**a**) parainfluenza, (**b**) human metapneumovirus, (**c**) rhinovirus, (**d**) adenovirus, (**e**) respiratory syncytial virus (RSV), (**f**) common human coronavirus (hCoV), (**g**) influenza virus, and (**h**) SARS-CoV-2. In each case, percent positivity (lines) corresponds to the left y-axis and total tests performed (shaded areas) corresponds to the right y-axis. CDC data were obtained from NREVSS for panels A-F (ending 2021), FluView for influenza virus, and COVID Data Tracker for SARS-CoV-2. AoU: *All of Us.*

Test positivity patterns from 2017 to 2022 matched CDC rates for most viruses (mean absolute error 5.89% positive tests per week for RV and 1.18–2.82 for all other viruses; Fig. 6). SARS-CoV-2, influenza virus, and RV had the highest percent positivity, and test positivity for most viruses followed expected seasonal patterns: PIV and RV test positivity showed two seasonal peaks per year (spring-dominant for PIV, fall-dominant for RV), while RSV, influenza virus, and hMPV had single overlapping winter peaks. SARS-CoV-2 test positivity rates matched expected variant waves (e.g., Alpha, Delta, and Omicron BA.1). Notable differences in the *All of Us* data include more week-to-week variability in virus positivity, undercounted positivity by ~ 10% during peak respiratory season for influenza virus and RSV, and less ADV positivity, relative to CDC data.

## Discussion

This study demonstrates significant variability in virus phenotyping performance across pathogens and algorithm parameters in large US EHR datasets, providing important insights for EHR-based respiratory virus research. By combining laboratory results, ICD codes, and medications, we improved case detection compared to any component alone – an approach particularly valuable in regions with less frequent laboratory testing such as the Southeast. The size differences between our cohorts (20,000–30,000 episodes for influenza virus and SARS-CoV-2 compared to 200-1,600 for the other viruses we considered) likely represent increased clinical suspicion and testing for common pathogens rather than true differences in disease burden^5^. The epidemiological patterns we identified matched national surveillance data, supporting the utility of this approach. These computable phenotypes offer a robust framework to conduct epidemiological, genomic, and clinical research on respiratory viruses using real-world healthcare data.

Phenotype performance varied substantially between viruses, across episode composition, and over time. While all phenotypes maintained high specificity, virus-specific ICD code sensitivity varied widely: higher for influenza (66.8% vs. 38–95% in published studies) and RSV (55.2% vs. 24%); moderate for SARS-CoV-2, ADV, hMPV, and hCoV (33–44%); and very low for PIV (8.3% vs. 14%) and RV (9.2% vs. 0%)^8,10,11,21,22^. Lower sensitivity rates for less common viruses likely reflect reduced clinical suspicion and greater reliance on nonspecific syndromic coding. Importantly, non-influenza, non-SARS-CoV-2 virus-specific ICD codes showed high PPV regardless of count (89.7–97.3%), whereas single ICD codes for SARS-CoV-2 and influenza demonstrated lower PPV (68.8–69.7%)^8,10,11,22^. In medication-inclusive cases, ICD codes had a higher PPV for both influenza virus and SARS-CoV-2 episodes. For SARS-CoV-2 specifically, antivirals alone were highly predictive of test results, although receipt of remdesivir, molnupiravir or nirmatrelvir was rare (4.57% of all episodes) during the study period. These performance differences highlight the necessity of selecting algorithm parameters based on the specific pathogen and research objective (i.e., a desire to maximize sensitivity for epidemiological surveillance or positive predictive value for genetic association studies).

The COVID-19 pandemic disrupted the seasonal transmission of most respiratory viruses, granting the opportunity to assess ICD code performance in unexpected settings and demonstrating the importance of evaluating code performance over time for seasonally variable diseases. hCoV ICD codes were used to identify concern for COVID-19, with diminished but persistent effects throughout the study period. Apart from rhinovirus, our phenotypes only rarely identified false positive episodes during the COVID-19 pandemic, mostly attributable to influenza virus episodes composed of a single ICD code. We suspect that these episodes reflect clinical concern for infection rather than true infection, and indeed, adjusting the influenza phenotype from any ICD code or medication to 2 + ICD codes or medications accompanied by an ICD code markedly reduced false positives and increased PPV.

Level-of-care analyses revealed patterns suggesting systematic detection biases toward higher acuity settings. Our phenotype identified a high frequency of infections at emergency and inpatient visits for RV, hMPV, RSV, ADV, PIV, and hCoV, compared to more outpatient visits for SARS-CoV-2 and influenza virus. These findings differ from established hospitalization rates: CDC estimates suggest that only 1–2% of medically-attended influenza cases require hospitalization, while COVID-19 hospitalization rates ranged between 2% and 68% over this study period, with temporal trends showing a decrease from ~ 50% in the early pandemic to 20% by July, 2022^23–27^. Moreover, prospective studies in adults have shown that hospitalization rates for other respiratory viruses are either similar or lower compared to influenza - the opposite of our results^3,6^. This discordance suggests that the *All of Us* computable phenotype oversamples high levels of care, particularly for non-influenza, non-SARS-CoV-2 viruses, likely due to at least three factors: the lack of cost-effective outpatient assays, an absence of specific therapeutic interventions that would justify multiplex testing costs in lower-acuity care settings, and the utility of identifying an etiology in the inpatient setting, where cessation of antibiotics or discharge are considerations.

Several limitations affect the utility of these methods. While strong correlation with CDC data supports the use of this method for comparative temporal studies, the low sensitivity we observed precludes absolute prevalence estimates. Although real-time surveillance would be feasible with other datasets, the current *All of Us* approach requires EHR data conversion to a common data model before periodic release.

Generalizability is also limited due to the representativeness of our sample. Despite broad national coverage, *All of Us* highly sampled some states (AZ, MA, WI, AL, PA, IL, MI, NY, MS, CA), while approximately half the US population was sampled at rates below 1 in 10,000. The program’s intentional oversampling of communities underrepresented in biomedical research also results in demographics that differ from the overall US population. Notably, this cohort showed a decrease in representation of persons of Asian, Middle Eastern or North African, and Native Hawaiian or Other Pacific Islander heritage. Other known impediments to generalizability compared to the US population include decreased representation of blind and deaf participants and challenges in linking EHRs from *All of Us* participants that joined outside a participant healthcare provider organization (indicated by a large gap between participants who agree to share EHR data and those with EHR data available)^28^.

Finally, this work faces challenges common to EHR-based research. These include labeling bias, implicit clinician biases which could be influenced by seasonality or patient demographics, and informed presence bias where EHR inclusion typically reflects illness rather than routine care^29,30^. The minimal representation of urgent care and post-acute care in our data further underscore the disconnected nature of healthcare in the US, and identifies a gap in infection detection in these cohorts.

Overall, this study presents computable phenotypes for identifying viral respiratory infections by combining virus-specific ICD codes, laboratory testing, and antiviral use in multi-center EHR data. This method reliably detected geographic and temporal patterns matching national surveillance, although severe infections are oversampled, and many mild infections are likely missed (supporting a need for expanded surveillance of non-influenza/non-SARS-CoV-2 pathogens in routine medical care)^31^. These phenotypes enable future studies of genetic susceptibility, environmental effects, and clinical outcomes research across both well-studied and understudied respiratory viruses. Given that *All of Us* broadly represents EHRs across the US, these approaches would likely be applicable to any US-based EHR-based dataset. Beyond respiratory viruses, this work serves as a foundation for the creation and validation of other computable phenotypes for episodic infectious diseases using EHR-based methods and emphasizes the importance of tailoring algorithm design and phenotype definition to specific research objectives.

### Methods

#### Data acquisition

All methods were carried out in accordance with *All of Us* program guidelines as well as local guidelines and regulations. Experimental protocols were approved by NHGRI Center for Precision Health Research. We analyzed data from the *All of Us* Research Program, which digitally enrolls participants aged 18 years and older across the United States^32^. *All of Us* is a large, diverse national cohort where participants contribute survey data, standardized physical measurements, biospecimens, and EHR data including billing codes, prescriptions, and laboratory results^23^. Participants provide consent to share health information, which includes physical measurements, surveys, genomic data, and EHRs. The informed consent and enrollment for all participants has been described, and specific Institutional Review Board approval is not required for Controlled Tier use of de-identified data, deemed nonhuman subjects research by the *All of Us* Institutional Review Board^32,33^. The program prioritizes recruitment of populations historically underrepresented in biomedical research^32^. This analysis used Controlled Tier data (C2022Q4R13) from the *All of Us* Researcher Workbench and was restricted to the 265,222 participants who had ICD codes, medications, or laboratory results in their EHR data between 1/1/1981 (the earliest influenza data available) and 7/1/2022. Data linkage, follow-up completeness, quality assessment, participant privacy, and community engagement are described in the *All of Us* Protocol^34^. This study meets all five of the CODE-EHR minimum framework standards for the use of structured health care data in clinical research, with one out of five standards meeting preferred criteria^35^. Participants’ demographic data were derived from the *All of Us* Researcher Workspace’s “person” table and “The Basics” survey.

## Phenotype development

We developed computable phenotypes for eight respiratory viruses: rhinovirus (RV), human metapneumovirus (hMPV), respiratory syncytial virus (RSV), adenovirus (ADV), SARS-CoV-2, parainfluenza (PIV), common human coronavirus (hCoV), and influenza virus. Patient encounters were identified in the EHR if they had at least one of the following: a virus-specific billing code (ICD-9-CM or ICD-10-CM), an antiviral indicated for the target pathogen, or a positive laboratory test. We identified virus-specific ICD codes and Logical Observation Identifiers Names and Codes (LOINC) laboratory results by searching for the virus name and related terms (e.g., “adenovirus” and “adenoviral pneumonia”). We excluded codes and results for zoonotic infections, vaccine-related events, and ICD codes for explicitly non-respiratory conditions (e.g., “ovine adenovirus”, “enteritis due to adenovirus”). We also excluded codes and results for pathogens sharing components of the virus name (e.g., “Haemophilus influenzae”). Laboratory data included nucleic acid amplification, antigen, and culture results. For influenza virus and SARS-CoV-2, we included antiviral medications (i.e., oseltamivir, zanamivir, and baloxavir for influenza virus; remdesivir, molnupiravir, and nirmatrelvir/ritonavir for SARS-CoV-2) given for more than one day. For oseltamivir and zanamivir, prescriptions were removed if the duration of treatment was greater than 6 days to exclude prophylaxis. Antivirals for other respiratory viruses were not included due to poor specificity or their restricted use for severe or immunocompromised cases.

We computed phenotypes for each infection episode by grouping related clinical events (Fig. 1a). An episode began with the first occurrence (t_0_) of any virus-specific component: a virus-specific ICD code, positive laboratory result, or qualifying antiviral prescription. All subsequent components within 90 days of t_0_ were considered part of the same episode, while events beyond 90 days initiated new episodes^5^. To capture false negatives, we included negative or indeterminate laboratory results from t_0_-5 days through the episode’s end. The per-episode occurrence of constituent components (ICD codes, positive laboratory results, and medications) were tallied (Fig. 1b). Counts for all virus-specific ICD codes, laboratory results, and medications used for phenotyping are provided (Tables S1-3). We de-duplicated row-level entries; categorized visit types into major categories (i.e., intensive care unit, inpatient, emergency room, urgent care, post-acute care, outpatient, and unknown); and reclassified laboratory results as positive, negative, or indeterminate (Table S5).

### Phenotype sensitivity analyses

#### Positive predictive value, specificity, and sensitivity calculations

Using nucleic acid amplification and virus culture test results from *All of Us* EHR data as a reference standard, we calculated the performance (PPV, sensitivity, and specificity) of ICD codes based on increasing counts of virus-specific ICD codes within each episode (e.g., multiple occurrences of J10.1, or combinations like J10.1 + J11 + 487). For a given threshold *N* (*n* = 0, *n* ≥ 1, *n* ≥ 2, *n* ≥ 3, *n* ≥ 4), we defined true positives as episodes with *N* virus-specific ICD codes and a positive test, false positives as episodes with N ICD codes and only negative tests, and false negatives as episodes with fewer than N ICD codes and a positive test.

For influenza virus and SARS-CoV-2, we additionally tested the performance of incorporating antiviral prescriptions with ICD codes. We first assessed specificity, sensitivity, and PPV in cases requiring both the specified ICD count and a prescription to be considered positive, or fewer than *N* ICD codes and no prescription to be considered negative. Then, we evaluated performance metrics (sensitivity, specificity, PPV, negative predictive value (NPV), and phi coefficient) across different combinations of ICD code thresholds and medication criteria for these two viruses.

#### Temporal and geographic analysis of Phenotype-Positive episodes

For any episode that met positivity criteria, we analyzed temporal patterns by calculating three-week moving averages of episode and constituent component counts from July 2017 (MMWR week 201726) through June 2022 (MMWR week 202225). Because hCoV PPV was lower than expected for non-influenza, non-SARS-CoV-2 viruses, and as hCoV ICD counts were disrupted during the COVID-19 pandemic, hCoV ICD codes after February 1, 2020 were excluded from the analysis (Figure S1). We assessed the geographic distribution of episode rates by three-digit ZIP code prefixes (zip3).

#### Level of care sensitivity analysis

Because of differences in testing and care by facility type and acuity, for each episode we identified the highest acuity encounter (from lowest to highest: outpatient, post-acute care, urgent care, emergency department, or inpatient) within a window spanning t_0_ – 7 days through t_0_ + 14 days. We chose this window after analyzing the distributions of visit timing for phenotype-related visits (those associated with virus-specific ICD codes, antivirals, or laboratory results) and all visits (Figure S4). Use of all visits within the selected window, rather than only phenotype-related visits, reduced the overall percentage of missing encounter data from 25.9 to 15.4%; across viruses and ICD counts, median missing encounter data decreased from 17.6% (IQR 7.6–28.3%) to 11.3% (IQR 2.2–17.8%; Table S6). Rarely (0.09-0.43%), phenotype-related maximum encounter acuity exceeded encounters within this time window (e.g., hospitalization occurring > 7 days prior to initial viral diagnosis, Figure S5). For PIV, hMPV, and RSV, manual review of these visits revealed that the associated encounter start date preceded the window period by a few days, and the phenotype component level of care was retained. We analyzed level-of-care patterns across viruses, stratifying by ICD codes and test positivity.

### Comparison with National surveillance data

To confirm the representativeness of our laboratory result data in *All of Us*, we compared the seasonal percent positivity and test volume of *All of Us* EHR laboratory results to data from three CDC/WHO sources: National Respiratory and Enteric Virus Surveillance System (NREVSS), COVID-19 Data Tracker, and the Global Influenza Surveillance and Response System (GISRS).

First, we assessed geographic coverage by comparing *All of Us* participant locations and testing rates to deduplicated NREVSS clinical laboratory results from 2017 to 2021^36^. *All of Us* EHR participant counts were visualized by aggregating data across three-digit ZIP code prefixes (zip3). Zip3 information was missing for 2/265,222 (0.00%). *All of Us* EHR participants (per 1,000 zip3 2020 Census population) and *All of Us* participants tested (per 1,000 *All of Us* participants with EHR data) were similarly aggregated by zip3 code. Zip3 regions with five or fewer *All of Us* participants were removed and classified as “No Data.” We obtained zip3 boundaries from US Census Bureau zip code tabulation areas, 2017 cartographic state boundaries from the Vega us-10 m.json dataset, and 2020 zip code tabulation area populations from the US Census Bureau.

Next, we assessed virus distribution by comparing proportions detected (% = N type / N total with known type) in *All of Us* to surveillance data from NREVSS and the GISRS^18–20^. These comparisons were limited to hCoV, influenza virus, and PIV, as these were the only viruses for which syndromic multiplex panels routinely report type-specific results.

To evaluate temporal patterns, we compared weekly test positivity data between *All of Us* and CDC surveillance from the first week of July, 2016 to the last week of June, 2022. For each virus, we calculated the percentage of positive tests for each *MMWR* reporting week. We plotted three-week moving averages for both percent positivity and total tests performed for *All of Us* and CDC data. We obtained CDC comparison data from NREVSS for non-SARS-CoV-2 viruses, additional influenza virus data from FluView, and SARS-CoV-2 data from the COVID Data Tracker^36–38^.

## Electronic supplementary material

Below is the link to the electronic supplementary material.

Supplementary Material 1

Supplementary Material 2

## Data Availability

The Community Workspace “Respiratory Virus Computable Phenotype” is available for all approved *All of Us* Controlled Tier users and includes all code used in this work (https://workbench.researchallofus.org/workspaces/aou-rw-ae307fda/respiratoryviralinfectionsinallofus/analysis).
